# Patterns of food parenting practices regarding junk food and sugary drinks among parent-child dyads

**DOI:** 10.1186/s12937-020-00610-3

**Published:** 2020-08-26

**Authors:** Jessica L. Thomson, Erin Hennessy, Alicia S. Landry, Melissa H. Goodman

**Affiliations:** 1 US Department of Agriculture, Agricultural Research Service, 141 Experiment Station Road, PO Box 225, Stoneville, MS 38776 USA; 2 Friedman School of Nutrition Science and Policy, Tufts University, 150 Harrison Avenue, Boston, MA 02111 USA; 3 Department of Family and Consumer Sciences, University of Central Arkansas, McAllister 112, 201 Donaghey Avenue, Conway, AR 72035 USA

**Keywords:** Junk food, Sugary drinks, Parenting practices, Legitimacy of parental authority, Dyads, Latent class analysis

## Abstract

**Background:**

Children’s food preference and intake patterns are affected by parental child feeding practices. The objective was to determine patterns of food parenting practices regarding junk food and sugary drinks (JS) and investigate their associations with demographic characteristics and dietary intake in a large cohort of parents and their children (12–17 years).

**Methods:**

Dyadic survey data from the cross-sectional, internet-based Family Life, Activity, Sun, Health, and Eating Study, conducted in 2014, were analyzed using latent class analysis to identify patterns of use for six JS parenting practices – negative emotions, restriction, monitoring, availability, modeling, and child involvement – based on parent and child report. Model covariates included self-reported parent and child sex, age (child only), body mass index category (based on height and weight), added sugars intake, and legitimacy of parental authority.

**Results:**

Based on 1657 parent-child dyads, five parenting practice patterns were identified representing different levels of practice use – Complete Influencers (28%; reference class), Indifferent Influencers (21%), Negative Influencers (20%), Minimal Influencers (18%), and Disagreeing Influencers (13%). Compared to older child dyads, younger child dyads were less likely to belong to Indifferent and Minimal Influencers (79 and 63% lower odds, respectively). Greater parent added sugars intake increased the odds of belonging to Indifferent and Negative Influencers (4 and 5% higher for every teaspoon increase, respectively) while greater child added sugars intake decreased the odds of belonging to Minimal Influencers (6% lower for every teaspoon increase). Compared to dyads with high scores, dyads with low child scores for legitimacy of parental authority regarding JS were 18 times as likely to belong to Disagreeing Influencers.

**Conclusions:**

The study findings suggest that parents utilize distinct patterns of feeding practices regarding JS ranging from use of many practices, use of some practices, to low use of any practice, with differential associations with parent and child intakes of added sugars. Counseling or intervening with parents to use a mix of structure practices, such as availability and modeling, to positively influence their child’s and possibly their own intake of sugary snacks and drinks may prove more efficacious than use of coercive control practices, such as negative emotions.

## Background

Consumption of added sugars, those added during processing or preparation of food and beverages, is linked to reduced diet quality; increased energy intake; increased risks for cardiovascular disease, obesity, and type 2 diabetes; and dental caries [1]. Because of public health concerns about the negative effects of added sugars consumption, particularly from beverages which account for almost half of added sugars consumption in the United States (US), the 2015–2020 Dietary Guidelines for Americans (DGA) recommend limiting added sugars intake to less than 10% of daily energy intake [1]. The World Health Organization concurred with the DGA recommendation, but further suggested a conditional recommendation of less than 5% of total energy intake from added sugars [2]. Recent evidence suggests that added sugars consumption is decreasing in US populations, nonetheless intake levels for both children and adults (14 and 17%, respectively) exceed recommendations [3, 4]. Junk foods, commonly defined as foods with low nutritive value that are high in added sugars, saturated fats or sodium, also have been linked to adverse health outcomes in both children and adults [5, 6]. A study examining consumption of junk food (including sugary drinks) in US children and adolescents found that junk food intake decreased by about 10% from 2003 to 2016 [7]. However, more than 70% of calories and over 90% of total sugars intake were derived from junk foods indicating intakes are still unacceptably high in children and adolescents [7]. Hence efforts that encourage and support children and adults to adopt healthy eating patterns that limit intake of junk food and sugary drinks are needed still.

Children’s food preferences, intake patterns, and weight status are affected by parental child feeding practices [8]. Given the relatively large number of practices that have been identified since the release of the Child Feeding Questionnaire in 2001 [9], researchers have proposed a content map that presents three overarching, higher order food parenting practice types – coercive control, structure, and autonomy [10]. Coercive control, a parent-centered strategy that satisfies parental goals and desires and may not consider children’s needs, such as pressure to eat, restriction and using food as a reward, generally have negative effects on children’s eating behaviors (e.g., aversion to healthful foods, tendency to overeat, increased snack consumption) [10]. Structure practices are exemplified by noncoercive parental control such as consistent enforcement of eating rules and boundaries and strategies to help children learn and maintain dietary behaviors [10]. Practices that demonstrate autonomy support for dietary behavior include nutrition education, child involvement, and encouragement [10]. Both structure and autonomy support practices generally are positively associated with healthful child dietary behaviors (e.g., lower intakes of snacks, higher fruit and vegetable consumption, increased acceptance of new foods) [10]. However, results pertaining to less healthful foods, such as sugary snacks and drinks, are contradictory with some studies finding more controlling or restrictive parenting practices associated with higher intakes [11] and others reporting lower associated intakes [12].

The contradictory results may be partially due to studying discrete relationships between food parenting practices and children’s diet. Parenting practices are often not used separately from one another as the use of some practices may influence the need for others [10]. Further, evidence suggests that food parenting practice use may be dependent on parent sex, and child age, sex, and weight status. Fathers of older versus younger children report greater child feeding responsibility and fathers use pressure to eat more often with their sons than daughters [13–15]. Conversely, mothers report greater child feeding responsibility, especially for younger children, and are more likely to use praise with their daughters than sons [14, 15]. In addition, restriction is more commonly used by parents of adolescents with overweight or obesity while pressure to eat is more commonly used by parents of adolescents with underweight/healthy weight [16]. Studying patterns of food parenting practices is necessary to determine which practices are used in combination, which are associated with more healthful dietary intake, and explore parent and child characteristics associated with specific patterns. Such knowledge is useful for designing interventions targeting effective strategies to guide children to make healthful dietary choices and improve dietary intake.

Much of the food parenting practice research has focused on specific practices with minimal attention given to elements that may affect children’s willingness to comply with them [10–12]. Whether children believe parents have a right to set rules about a particular domain of their life – a concept known as the legitimacy of parental authority – partially governs the choice to obey or not obey [17]. This is an important concept to study as children age because they are inclined to desire more autonomy and less parental control or authority. Associations among legitimacy of parental authority, food parenting practices, and dietary intake are largely understudied, especially in terms of parent-child dyadic relationships.

In this paper, publicly available data from the Family Life, Activity, Sun, Health and Eating (FLASHE) Study [18] were analyzed to identify subtypes of parent-child dyads that exhibited similar patterns of food parenting practices. FLASHE was designed to examine psychosocial, generational (parent-child), and environmental correlates of cancer preventive behaviors from an individual and dyadic perspective [19]. All three types of parenting practices were measured – coercive control, structure, and autonomy support – and fathers, under-represented in the parenting practice literature [15], were purposively included [19]. A dyadic approach allowed for exploration of interdependence between parent- and child-reported food parenting practices. Hence, the objectives of this paper– to determine patterns of food parenting practices using a person-orientated approach (latent class analysis) and to investigate associations among patterns and parent and child demographic characteristics and dietary intake – address two gaps in the literature. It was hypothesized that multiple patterns of food parenting practices exist and that they would be differentially associated with both parent and child demographic characteristics and dietary intake.

## Methods

### Data source and sample

Survey data from FLASHE, a cross-sectional, Internet-based study conducted from April to October 2014 and sponsored by the National Cancer Institute (NCI), were used for these analyses [19]. An online consumer opinion panel was used to recruit eligible parent-child dyads and surveys were administered via the web. Eligibility criteria included: at least 18 years old; at least one child aged 12–17 years living at least 50% of the time in the household; agreed to be contacted for study participation. One eligible child was randomly selected from eligible households. The household sample was created using balanced sampling and is similar to the general US population for sex, income, age, household size, and region [20]. A total of 1945 dyads (parent-caregiver and child) were enrolled. Study participation involved completion of six web surveys, three by the parent and three by the child. FLASHE was approved by the United States (US) Government’s Office of Management and Budget, the NCI Special Studies Institutional Review Board, and Westat’s Institutional Review Board. Additional details on study methods have been published elsewhere [20].

### Demographic and anthropometric characteristics

For analytic purposes, child age was categorized into two groups, representing early adolescence (12–14 years) and middle adolescence (15–17 years). For parents and children, race/ethnicity were ascertained with two questions that were combined to create four categories – Hispanic, non-Hispanic black or African American only, non-Hispanic white only, and non-Hispanic other. Responses for parents’ education level included less than a high school degree, a high school degree or General Educational Development (GED) certification, some college but not a college degree, and a 4-year college degree or higher. Responses for parents’ marital status included married, divorced, widowed, separated, never married, and member of an unmarried couple. Divorced, widowed, and separated were combined into a single category in the public use dataset. Responses for household income (parent survey) included nine options ranging from $0–$9999 to $200,000 or more that were dichotomized to $0–$99,999 and $100,000 or more in the public use dataset. Using parent and child self-reported values, BMI was calculated as weight (kg) divided by height (m^2^). For parents, body weight was classified as underweight (BMI < 18.5), healthy weight (18.5 ≤ BMI < 25), overweight (25 ≤ BMI < 30), and obesity (BMI ≥ 30). For children, body weight was classified based on Centers for Disease Control and Prevention’s sex-specific 2000 BMI-for-age growth charts as underweight (BMI < 5th percentile), healthy weight (5th percentile ≤BMI < 85th percentile), overweight (85th percentile ≤BMI < 95th percentile), and obesity (BMI ≥ 95th percentile). For analytic purposes, body weight categories were collapsed to underweight/healthy weight and overweight/obesity.

### Dietary intake

The FLASHE dietary screener (27 items) was used to capture parent and child dietary intake frequency for foods and beverages that have remained of interest in dietary guidance in the US [21]. Complete screener wording can be found on the FLASHE website [19]. Response options, based on the past 7 days, included no consumption, 1–3 times/week, 4–6 times/week, 1 time/day, 2 times/day, and ≥ 3 times/day. The responses 1–3 times/week and 4–6 times/week were converted to daily intake frequencies by dividing the median by 7 resulting in values of 0.29 an 0.71 times/day, respectively. The response ≥3 times/day was coded as 3 times/day. Daily frequencies of each food and beverage group were calculated by summing individual item frequencies based on groupings used in previous studies [22]. These groupings included fruits, vegetables, fruits and vegetables combined (with and without fried potatoes), dairy, added sugars (total and sugary drinks only), and whole grains. Specific foods and beverages related to added sugars intake included sugary cereal, candy and chocolate, chips, cookies, cake, frozen desserts, sweetened fruit drinks, regular soda, sports drinks, and energy drinks. Using programs developed to compare responses from the National Health and Nutrition Examination Survey dietary screener (similar to the FLASHE dietary screener) with the What We Eat in America 24-h dietary recall data [21], daily teaspoons of added sugars were estimated from daily frequencies [23]. For the current study, dyads were included in the analyses if they had non-missing data on parent and child daily intakes (teaspoons) of added sugars.

### Junk food and sugary drinks (JS) parenting practices and legitimacy of parental authority

Six JS parenting practices were measured with one item each and represented two types of coercive control practices – negative emotions (allow JS when had bad day) and restriction (parent decides JS amount); three types of structure practices – monitoring (do not eat too much JS), availability (do not buy JS), and modeling (avoid eating JS when child around); and one type of autonomy support practice – child involvement (decide together JS amount). Additionally, legitimacy of parental authority regarding JS (JS-LPA) was measured with one item (okay to make rules about JS). On the surveys, junk foods were defined as foods that are high in calories and usually have added sugars and fat and include candy, cookies, potato chips, French fries, etc. Sugary drinks were defined as regular soda, sports drinks, fruit drinks, sweetened teas, and other drinks with added sugars. The items were taken or modified from valid and reliable instruments using cognitive testing; source information and full survey wording can be found on the FLAHSE website [19] and in Supplementary Table 1, Additional file 1. Items were included on parent and child diet surveys and responses ranged from strongly disagree (1) to strongly agree (5). For analytic purposes, responses were dichotomized as strongly disagree to neither agree nor disagree (1–3) and agree to strongly agree (4–5).

### Statistical analysis

Statistical analyses were performed using SAS® software, version 9.4 (SAS Institute Inc., Cary, NC). The statistical significance level was set at 0.05. Dietary survey weights for parent and child cohorts were used for computing descriptive statistics for demographic characteristics, BMI, added sugars intake, JS parenting practices, and JS-LPA. Sample weights were not used for correlation analysis or latent class analysis because no dyadic analysis weights were provided (population control totals cannot be easily defined) [24]. Each parent was linked to only one child and child was linked to only one parent. Parents and children were asked the same questions to facilitate dyadic analysis. Dyadic analysis was performed using the parent-child dyad identifier. Comparisons between the analytic and excluded dyads on demographic characteristics were performed using chi square tests of association. To confirm associations among JS parenting practices, Spearman rank correlation coefficients (r_s_) were computed because variables were measured on an ordinal scale and some distributions were skewed. Correlation coefficients’ strength was based upon Cohen’s recommendations (weak < 0.30, moderate =0.30–0.49, and strong ≥0.50) [25].

To identify subtypes of parent-child dyads that exhibited similar patterns of food parenting practices, PROC LCA [26] was used to conduct latent class analysis based on 12 indicators (six parent- and six child-reported JS parenting practices) and step recommendations by Bray et al. [27]. Model selection was conducted using one through six class solutions. Information criteria, entropy, and interpretability of each latent class solution were used to select the appropriate number of classes. Entropy refers to the certainty of model selection with values near one indicating high certainty. Interpretability is based on how clearly classes are distinguished from one another based on item-response probabilities. Item-response probabilities represent the probability of a reporting agreement with a specific parenting practice given membership in a specific latent class. Missing data on parenting practice indicators were accounted for using full-information maximum likelihood estimation. The selected latent class model was re-fit with child age group (12–14 and 15–17 years) and parent and child sex, BMI category, added sugars intake, and JS-LPA included as covariates to produce posterior probabilities. The addition of covariates resulted in a set of regression coefficients, representing the increase in odds of belonging to a class relative to a reference class, corresponding to each covariate attribute. Maximum-probability assignment was used to assign dyads to the class for which they had the highest posterior probability of membership which allowed for descriptive (not inferential) comparisons between classes.

## Results

Of the 1945 parent-child dyads enrolled in the FLASHE study, 1657 (85%) were included in the present analyses. Statistically significant differences in demographic characteristics were not found between analytic and excluded dyads except that proportionally more parents were married and less were divorced/widowed/separated in analytic as compared to excluded dyads and proportionally more 14 year old and less 15 year old adolescents were in analytic as compared to excluded dyads. Characteristics and measures of the parent-child dyads in the analytic sample are presented in Table 1. The majority of parents were between 34 and 59 years of age (87%), female (57%), white (60%), educated (53% with at least a 4-year college degree), and married (79%). For children, approximately half were female (49%) and white (55%). Mean parent BMI was in the overweight range, while mean child BMI percentile was in the healthy weight range. Mean parent added sugars intake was higher than child added sugars intake (18 and 16 teaspoons, respectively). Mean parent-reported parenting practices and LPA were generally higher than child-reported means.

**Table 1:** Parent and child characteristics and measures^a^ (*N* = 1657 dyads)

Parent	Number	Percent
Age (years)		
18–34	188	10.2
34–44	713	42.8
45–59	694	43.8
60+	47	3.2
Sex		
Male	421	43.5
Female	1221	56.5
Race/Ethnicity		
Hispanic	117	16.1
NH black/African American	277	11.8
NH white	1138	60.0
NH other	95	12.2
Education Level		
< High school	21	1.1
High School/GED	272	14.9
Some college	577	30.7
≥ 4-yr college degree	767	53.2
Marital Status		
Married	1179	78.9
Divorced/widowed/separated	200	8.9
Never married	157	7.8
Unmarried couple	92	4.4
Household Income		
$0–$99.999	1287	70.9
$100,00+	337	29.1
**Parent**	**Mean**	**SD**
BMI^b^	27.7	7.12
Added sugars intake (tsp)	18.0	10.36
Added sugars from beverage intake (tsp)	10.4	12.05
Parenting Practice (JS)^c^		
CC: negative emotions	2.4	1.14
CC: restriction	3.5	1.16
S: monitoring	3.6	1.24
S: availability	3.5	1.19
S: modeling	3.2	1.22
AS: child involvement	3.2	1.17
LPA (JS)^c^	4.1	0.94
**Child**	**n**	**%**
Age (years)		
12	219	13.1
13	326	19.4
14	276	16.8
15	288	16.2
16	326	21.2
17	202	13.4
Sex		
Male	810	50.7
Female	823	49.3
Race/Ethnicity		
Hispanic	160	16.2
NH black/African American	272	13.8
NH white	1037	54.9
NH other	152	15.1
School Type		
Public	1379	84.5
Private	117	7.0
Home	104	6.4
Other	36	2.2
**Child**	**Mean**	**SD**
BMI percentile^b^	61.1	29.27
Added sugars intake (tsp)	16.0	7.65
Added sugars from beverage intake (tsp)	6.7	7.01
Parenting Practice (JS)^c^		
CC: negative emotions	2.4	1.19
CC: restriction	3.2	1.26
S: monitoring	3.2	1.32
S: availability	3.4	1.24
S: modeling	3.1	1.26
AS: child involvement	3.1	1.25
LPA (JS)^c^	3.5	1.16

*NH* non-Hispanic, *SD* standard deviation, *BMI* body mass index, *tsp.* teaspoon, *JS* junk food and sugary drinks, *CC* coercive control, *S* structure, *AS* autonomy support, *LPA* legitimacy of parental authority

^a^Percentages and means are weighted with dietary survey weights for parents and children

^b^Based on self-reported height and weight

^c^Scale range is 1 (strongly disagree) to 5 (strongly agree)

Correlations among JS parenting practices are presented in Table 2. Correlations among parent-reported practices ranged from non-existent (r_s_ = − 0.06 between negative emotions and monitoring and r_s_ = 0.06 between negative emotions and child involvement) to strong (r_s_ = 0.60 between restriction and monitoring). Similarly, correlations among child-reported practices ranged from non-existent (r_s_ = − 0.02 between negative emotions and restriction) to strong (r_s_ = 0.66 between restriction and monitoring). Correlations between parent- and child-reported practices ranged from moderate (r_s_ = 0.43 monitoring and child involvement) to strong (r_s_ = 0.57 negative emotions).

**Table 2:** Correlations^a^ among parenting practices regarding junk food and sugary drinks

Parenting Practice	CC: NE	CC: RS	S: MN	S: AV	S: MD	AS: CI
*Parent-Reported*						
CC: negative emotions	1.00	−0.09	− 0.06	− 0.25	−0.10	0.06
CC: restriction		1.00	0.60	0.22	0.25	0.44
S: monitoring			1.00	0.17	0.24	0.32
S: availability				1.00	0.51	0.19
S: modeling					1.00	0.35
AS: child involvement						1.00
*Child-Reported*						
CC: negative emotions	1.00	−0.02	0.03	−0.15	−0.07	0.05
CC: restriction		1.00	0.66	0.26	0.31	0.59
S: monitoring			1.00	0.19	0.28	0.49
S: availability				1.00	0.59	0.26
S: modeling					1.00	0.39
AS: child involvement						1.00
*Between Parent and Child*						
CC: negative emotions	0.57					
CC: restriction		0.51				
S: monitoring			0.43			
S: availability				0.47		
S: modeling					0.52	
AS: child involvement						0.43

*CC* coercive control, *NE* negative emotions, *RS* restriction, *S* structure, *MN* monitoring, *AV* availability, *MD* modeling, *AS* autonomy support, *CI* child involvement

^a^Spearman rank correlation coefficients; correlations ≥ |0.09| significant at *p* < 0.001; |0.05| ≤ correlations < |0.09| significant at *p* < 0.04

### Latent class analysis

**Fig. 1:**
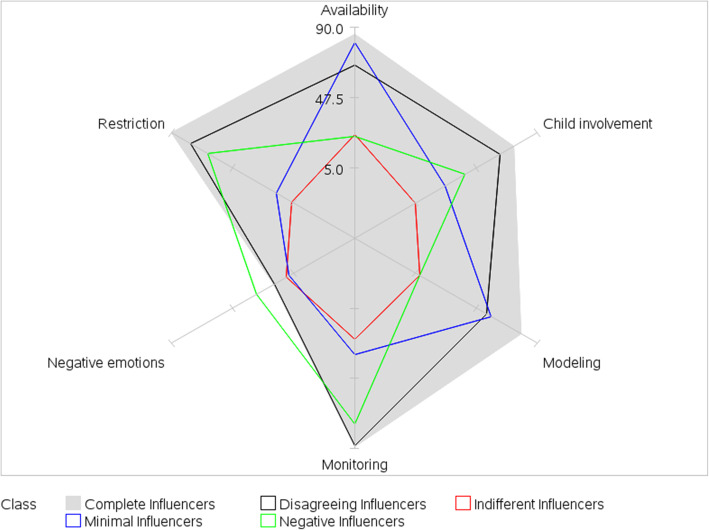
Latent class item-response probabilities for parent-reported parenting practices regarding junk food and sugary drinks. Spokes represent item-response probabilities converted to percentages. Item-response probabilities represent the probability of agreement with a specific parenting practice given membership in a specific latent class. Complete Influencers (28% of dyads) = high probabilities for all parent-reported JS parenting practices, except negative emotions. Indifferent Influencers (21% of dyads) = low probabilities for all parent-reported JS parenting practices. Negative Influencers (20% of dyads) = high probabilities of parent-reported negative emotions, restriction, and monitoring; and low probabilities of parent-reported availability and modeling. Minimal Influencers (18% of dyads) = high probabilities of parent-reported availability; low probabilities of parent-reported negative emotions, restriction, monitoring, and child involvement. Disagreeing Influencers (13% of dyads) = high probabilities of parent-reported restriction, monitoring, availability, and child involvement

**Fig. 2:**
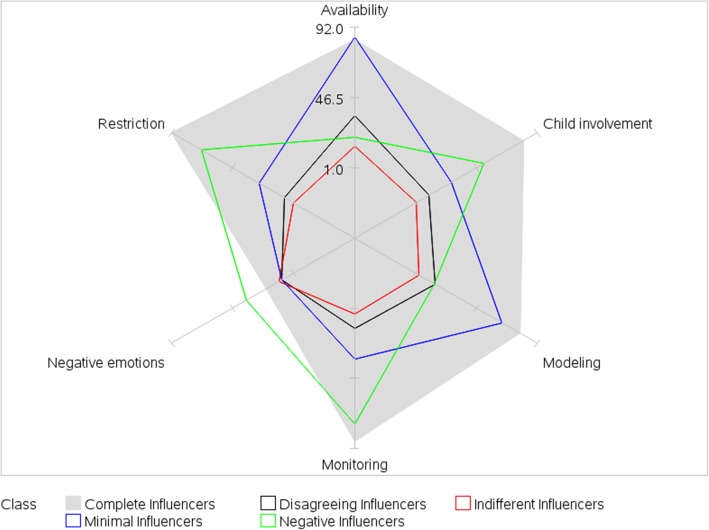
Latent class item-response probabilities for child-reported parenting practices regarding junk food and sugary drinks. Spokes represent item-response probabilities converted to percentages. Item-response probabilities represent the probability of agreement with a specific parenting practice given membership in a specific latent class. Complete Influencers (28% of dyads) = high probabilities for all child-reported JS parenting practices, except negative emotions. Indifferent Influencers (21% of dyads) = low probabilities for all child-reported JS parenting practices. Negative Influencers (20% of dyads) = high probabilities of child-reported negative emotions, restriction, and monitoring; and low probabilities of child-reported availability and modeling. Minimal Influencers (18% of dyads) = high probabilities of child-reported availability and modeling; and low probabilities of child-reported negative emotions, restriction, monitoring, and child involvement. Disagreeing Influencers (13% of dyads) = low probabilities for all child-reported JS parenting practices

Model fit statistics (see Supplementary Table 2, Additional file 2) supported a five-class model and classes were interpreted and labeled based on item-response probabilities (Figs. 1 and 2; Supplementary Table 3, Additional file 3). The term “influencer” was used to describe all five classes because it reflects the concept that parents are attempting to influence their children’s dietary intake through the use of parenting practices. Class 1 was labeled Complete Influencers and represented 28% of dyads; members were characterized by high probabilities for all parent- and child-reported JS parenting practices, except negative emotions. Class 2, labeled Indifferent Influencers, represented 21% of the dyads and members were characterized by low probabilities for all parent- and child-reported JS parenting practices. Class 3, labeled Negative Influencers, represented 20% of the dyads and members were characterized by high probabilities of parent- and child-reported negative emotions (relative to marginal probabilities of 18 and 19% for parent and child, respectively), restriction, and monitoring; and low probabilities of parent- and child-reported availability and modeling. Child involvement did not help define this class. Class 4, labeled Minimal Influencers, represented 18% of the dyads and members were characterized by high probabilities of parent- and child-reported availability; low probabilities of parent- and child-reported negative emotions, restriction, monitoring, and child involvement; and disagreement on modeling (moderate parent-reported and high child-reported probabilities). Class 5, labeled Disagreeing Influencers, represented 13% of the dyads and members were characterized by high probabilities of parent-reported restriction, monitoring, availability, and child involvement; and low probabilities for all child-reported JS parenting practices. Moderate probabilities for parent-reported negative emotions and modeling disagreed with low child-reported probabilities.

Odds ratios and corresponding 95% confidence intervals for covariate effects are presented in Table 3. Significant effects were found for child age group, parent and child added sugars intake, and parent- and child-reported JS-LPA. For all comparisons, the reference class was Complete Influencers (i.e., odds of belonging to specific class as compared to odds of belonging to Complete Influencers). Compared to older child dyads, younger child dyads had 79 and 63% lower odds of belonging to Indifferent and Minimal Influencers, respectively. The odds of belonging to Indifferent and Negative Influencers were 4 and 5%, respectively, higher for every teaspoon increase in parent intake of added sugars while the odds of belonging to Minimal Influencers were 6% lower for every teaspoon increase in child intake of added sugars. Compared to dyads with high parental scores for JS-LPA, dyads with low parental scores had 17.6 and 25.7 times the odds of belonging to Minimal and Indifferent Influencers, respectively. Similarly, compared to dyads with high child scores for JS-LPA, dyads with low child scores had 2.4, 5.1, 11.2 and 18.3 times the odds of belonging to Negative, Minimal, Indifferent, and Disagreeing Influencers, respectively.

**Table 3:** Odds ratios and 95% confidence intervals for parent and child characteristics by latent class membership^a^

Characteristic	Indifferent Influencers			Negative Influencers			Minimal Influencers			Disagreeing Influencers			
	OR	95% CI		OR	95% CI		OR	95% CI		OR	95% CI		P
Child age group (Y:O)	**0.21**	**0.13**	**0.33**	1.08	0.71	1.64	**0.37**	**0.23**	**0.58**	0.64	0.40	1.03	< 0.001
Parent sex (male:female)	0.92	0.53	1.59	0.70	0.43	1.16	1.25	0.72	2.18	1.19	0.69	2.07	0.335
Child sex (male:female)	0.71	0.44	1.13	1.15	0.77	1.73	0.60	0.37	0.97	0.78	0.49	1.25	0.109
Parent BMI (OwOb:UwHw)^b^	1.46	0.91	2.36	1.08	0.71	1.63	0.93	0.58	1.48	1.40	0.87	2.27	0.246
Child BMI (OwOb:UwHw)^c^	0.84	0.50	1.43	1.36	0.87	2.12	0.99	0.58	1.68	1.19	0.71	1.97	0.452
Parent added sugars intake (tsp)	**1.04**	**1.01**	**1.07**	**1.05**	**1.02**	**1.08**	0.99	0.96	1.03	0.99	0.96	1.02	< 0.001
Child added sugars intake (tsp)	0.98	0.94	1.02	1.01	0.98	1.04	**0.94**	**0.90**	**0.98**	1.00	0.96	1.04	0.012
PR JS-LPA (low:high)	**25.73**	**12.02**	**55.09**	2.11	0.87	5.13	**17.60**	**8.17**	**37.89**	0.25	0.04	1.46	< 0.001
CR JS-LPA (low:high)	**11.17**	**6.65**	**18.78**	**2.35**	**1.40**	**3.95**	**5.11**	**2.93**	**8.91**	**18.34**	**10.87**	**30.94**	< 0.001

*OR* odds ratio, *CI* confidence interval, *Y* younger (12–14 years), *O* older (15–17 years), *BMI* body mass index, *OwOb* overweight/obesity, *UwHw* underweight/healthy weight, *tsp.* teaspoon, *PR* parent-reported, *JS-LPA* legitimacy of parental authority regarding junk food and sugary drinks, *CR* child-reported

^a^Reflects associations between latent class membership and dyad characteristic; Complete Influencers is reference class; second

characteristic in pair is reference characteristic (e.g. males compared to females). Bolded values indicate significant odds ratios

^b^OwOb defined as BMI ≥ 25 kg/m^2^; UwHw defined as BMI < 25 kg/m^2^

^c^OwOb defined as BMI ≥ 85th percentile; UwHw defined as BMI < 85th percentile

Parent and child characteristics of the five latent classes using maximum-probability assignment are presented in Table 4. Proportionally, more younger child dyads were in Complete and Negative Influencers as compared to Indifferent and Minimal Influencers. Minimal Influencers had the lowest proportion of male child dyads. Complete and Minimal Influencers had the lowest proportions of dyads with parental overweight/obesity while Negative and Disagreeing Influencers had the highest proportions of dyads with child overweight/obesity. Complete and Disagreeing Influencers had the lowest proportions of dyads with low scores for parent-reported JS-LPA. For all classes, Complete Influencers had the lowest proportion of dyads with low scores for child-reported JS-LPA. Minimal and Negative Influencers had the lowest and highest, respectively, mean intakes of added sugars for parent and child.

**Table 4:** Parent and child characteristics of latent classes using maximum-probability assignment

Characteristic	Complete Influencers		Indifferent Influencers		Negative Influencers		Minimal Influencers		Disagreeing Influencers	
	n	%	n	%	n	%	n	%	n	%
Overall prevalence	401	29.6	263	19.4	239	17.7	224	16.5	227	16.8
Child age group (12–14 years)	251	62.6	69	26.2	155	64.9	89	38.7	120	52.9
Parent sex (male)	103	25.7	74	28.1	60	25.1	61	26.5	56	24.7
Child sex (male)	216	53.9	123	46.8	141	59.0	92	40.0	116	51.1
Parent BMI (OwOb)^a^	232	57.9	182	69.2	148	61.9	128	55.7	146	64.3
Child BMI (OwOb)^b^	105	26.2	65	24.7	81	33.9	59	25.7	73	32.2
PR JS-LPA (low)	13	3.2	165	62.7	21	8.8	103	44.8	4	1.8
CR JS-LPA (low)	54	13.5	190	72.2	59	24.7	112	48.7	165	72.7
	**Mean**	**SD**	**Mean**	**SD**	**Mean**	**SD**	**Mean**	**SD**	**Mean**	**SD**
Parent added sugars intake (tsp)	15.4	10.82	18.9	10.55	21.5	12.28	14.0	7.74	15.0	7.86
Child added sugars intake (tsp)	15.5	8.15	16.7	7.60	18.4	8.17	13.5	5.02	16.1	7.47

*BMI* body mass index, *OwOb* overweight/obesity, *PR* parent-reported, *JS-LPA* legitimacy of parental authority regarding junk food and sugary drinks, *CR* child-reported, *SD* standard deviation, *tsp*. teaspoon

^a^OwOb defined as BMI ≥ 25 kg/m^2^

^b^OwOb defined as BMI ≥ 85th percentile

## Discussion

The purpose of this study was to determine patterns of parent- and child-reported JS parenting practices and to investigate their associations with demographic characteristics and dietary intake. A continuum of five patterns emerged representing parents and children who reported almost complete use of the six JS parenting practices (Complete Influencers), use of some of the practices (Negative Influencers, Minimal Influencers, and Disagreeing Influencers), and low use of the practices (Indifferent Influencers). Significant associations among the five patterns and child age, parent and child intakes of added sugars, and parent and child agreement with JS-LPA were observed. In a study conducted with Hispanic children 8–16 years of age, the most prevalent cluster had high scores for the three parenting practices measured – rules and limits, monitoring, and pressure to eat – similar to the most prevalent class, Complete Influencers, in the present study [28]. In both studies, the most prevalent cluster/class contained a mix of both favorable (structure and autonomy) and less favorable (coercive control) types of parenting practices in terms of associations with child dietary outcomes. Another cluster had low scores for the three parenting practices measured resembling the Indifferent Influencers in the present study [28]. Taken together, the results indicate that there exist sets of parents who either use a mix of JS parenting practices, both favorable and unfavorable, or do not attempt to influence their children’s JS intake.

The present study’s finding that dyads with older children are more likely to belong to Indifferent or Minimal Influencers as compared to Complete Influencers confirms results from an investigation of family perceptions of child feeding practices in which older versus younger siblings reported significantly less restriction and parents reported feeling less responsibility for feeding their older versus younger children [15]. The decreasing parental feeding responsibility may be the result of children’s desire for more autonomy in decision-making as they age and parents’ willingness to grant it. This shifting of responsibilities from parent to child is mirrored in the present study’s finding that Indifferent and Minimal Influencers, the classes with the lowest number of reported parenting practices used, were more likely to contain dyads with low parental and child scores for JS-LPA as compared to Complete Influencers. However, the lower reported child intake of added sugars observed in Minimal Influencers as compared to Indifferent Influencers suggests that the use of at least some structure practices (e.g., availability and modeling) are helpful for reducing intake of added sugars in older children.

The existence of Disagreeing Influencers in the present study is in line with other research that has found a lack of correspondence between parents’ and their children’s perceptions about parental feeding practices [15]. In particular, disagreement between parent- and child-report on restriction may be associated with the more covert form of this parenting practice [29] making it less likely that children are aware of their parents’ restrictive feeding practices [15]. Disagreement between parent and child on use of parenting practices in the present study also is mirrored in the disagreement between parent and child on JS-LPA. Disagreeing Influencers had the largest disparity in proportions of parents and children agreeing with JS-LPA (99% versus 26%, respectively). In a study investigating associations between parenting styles and parent-adolescent relationship factors, both youth-reported agreement with legitimacy of parental authority and level of conflict intensity differed by parenting style [30]. Thus, it is possible that the parenting style(s) of Disagreeing Influencers differed from the other classes. Given the novelty of these relationships, future studies should explore associations among parenting practices, legitimacy of parental authority, and parenting styles.

No studies reporting associations between the use of JS parenting practices and parent intake of added sugars could be found in the literature. Parents who report using a mix of JS parenting practices may be more aware of their food habits resulting in lower reported added sugars intake. The association between use of coercive control parenting practices and higher parental intake of added sugars also has not been reported in the literature. Coercive control practices generally are considered to negatively impact children’s eating behaviors (e.g., aversion to healthful foods, tendency to overeat, increased snack consumption) [10] and results from the present study suggest that this negative impact extends to parental dietary behaviors as well. If these results are confirmed by other studies, then nutrition interventions designed to positively influence the use or non-use of food parenting practices may result in synergistic improvements in dietary behaviors for children and their parents.

The present study’s findings of the association between use of some structure practices (Minimal Influencers = availability and modeling) in the absence of coercive control and autonomy support practices and lower intake of added sugars in children is intriguing. In the study conducted with Hispanic children, as compared to the cluster with low scores for all three parenting practices, the cluster with high scores for all three practices and the one with a high score for pressure to eat (but low scores for rules and limits and monitoring) had increased odds of having children with high obesogenic dietary intake (e.g., snack foods, sweets, sugary drinks) [28]. In a pediatric clinic based study conducted with children with overweight or at risk for overweight, higher use of parental monitoring was associated with lower child intake of sugary drinks, but use of restriction was not associated with child intake of sugary drinks [31]. In another study conducted with Dutch adolescents, higher scores for perceived parenting practices involving specific rules about sugary drink consumption were associated with decreased sugary drink consumption [12]. Collectively, results suggest that use of at least some structure practices, such as parental monitoring, availability and rule-setting, could be more helpful for reducing children’s consumption of added sugars than coercive control practices.

Several study limitations bear mentioning. All data were self-reported and hence subject to bias. Distributions of the JS parenting practice negative emotions and of JS-LPA were skewed but this did not adversely affect the analyses because variables were analyzed dichotomously. However, dichotomizing measures of parenting practices and legitimacy of parental authority limited the ability to examine class differences in scales of agreement. Although the inclusion of multiple health behaviors is a strength, measuring constructs with limited numbers of items may not have provided comprehensive assessments. As is often the case with broad-scope surveys, measures needed to be brief to reduce participant burden. While the household sample is similar to the general US population for sex, income, age, household size, and region, it may not be generalizable to specific subgroups (e.g., racial/ethnic, low education) in the US population.

## Conclusions

The study findings suggest that parents utilize distinct patterns of feeding practices related to JS ranging from use of many practices of all three types (coercive control, structure, and autonomy support), use of some structure practices, use of mostly coercive control practices, low use of any practice, and parent-child disagreement on use of practices. Patterns that contain a mix of structure and/or autonomy support type practices are associated with lower added sugars intake in both parents and their children while patterns that contain mostly coercive control type practices or low use of any practice are associated with higher added sugars intake in both parents and their children. These results suggest that counseling or intervening with parents to use a mix of structure and/or autonomy support practices, such as availability and modeling, to positively influence their child’s and possibly their own intake of sugary snacks and drinks may prove more efficacious than use of coercive control practices, such as negative emotions. Finally, the study’s findings suggest that there is a shift in belief about feeding responsibility between parent and child as children age. Parental use of a few structured feeding practices with their older children may add to parents’ legitimacy and increase their children’s willingness to obey rules, which in turn, may positively influence children’s intake of added sugars.

## Supplementary information

**Additional file 1: Supplementary Table 1.** Parenting practice items regarding junk food and sugary drinks included in the FLASHE surveys. Table contains construct type and survey item wording for the 12 parenting practices (6 parent-reported and 6 child-reported) included in the FLASHE surveys.

**Additional file 2: Supplementary Table 2.** Overall fit statistics for latent class models of parent- and child-reported JS parenting practices. Table contains model fit statistics for the latent class analysis using 1–6 latent classes and based on the 12 parenting practices (6 parent-reported and 6 child-reported) included in the FLASHE surveys.

**Additional file 3: Supplementary Table 3.** Latent class prevalence and item-response probabilities for parent- and child-reported JS parenting practices. Table contains the class prevalence and item-response probabilities for the 5-class solution using latent class analysis on the 12 parenting practices (6 parent-reported and 6 child-reported) included in the FLASHE surveys.

## Data Availability

The datasets analyzed during the current study are available on the National Cancer Institute’s FLASHE website, Data Resource Page (https://cancercontrol.cancer.gov/brp/hbrb/flashe-terms.aspx).

## Abbreviations

BMI: Body mass index
DGA: Dietary guidelines for americans
FLASHE: Family life, activity, sun, health and eating
JS: Junk food and sugary drinks
JS-LPA: Legitimacy of parental authority regarding junk food and sugary drinks
NCI: National Cancer Institute
US: United States
