# Supplementary material for: Patterns of food parenting practices regarding junk food and sugary drinks among parent-child dyads
Source: Nutr J. 2020 Aug 26;19:91. doi: 10.1186/s12937-020-00610-3 (PMC7448982; doi:10.1186/s12937-020-00610-3)
Supplement: Supplementary file 1 — Additional file 1: Supplementary Table 1. Parenting practice items regarding junk food and sugary drinks included in the FLASHE surveys. Table contains construct type and survey item wording for the 12 parenting practices (6 parent-reported and 6 child-reported) included in the FLASHE surveys. [file 12937_2020_610_MOESM1_ESM.docx]

| **Supplementary Table 1** Parenting practice items regarding junk food and sugary drinks included in the FLASHE surveys | | |
| --- | --- | --- |
| **Construct** | **Practice** | **Item** |
| *Parent survey* | | |
| Coercive control | Negative emotions | If my teenager has a bad day, I let him/her have junk food or sugary drinks to feel better. |
| Coercive control | Restriction | I have to make sure that my teenager doesn't eat too much junk food or drink too many sugary drinks. |
| Structure | Monitoring | I decide how much junk food or sugary drinks my teenager can have. |
| Structure | Availability | I don't buy a lot of junk food or sugary drinks for my teenager. |
| Structure | Modeling | I try to avoid eating junk food or drinking sugary drinks when my teenager is around. |
| Autonomy support | Child involvement | My teenager and I decide together how much junk food and sugary drinks he/she can have. |
| *Child survey* | | |
| Coercive control | Negative emotions | If I've had a bad day, my parent(s) let me have junk food or sugary drinks to make me feel better. |
| Coercive control | Restriction | My parent(s) have to make sure I don't eat too much junk food or drink too many sugary drinks. |
| Structure | Monitoring | My parent(s) decide how much junk food or sugary drinks I can have. |
| Structure | Availability | My parent(s) don't buy a lot of junk food or sugary drinks for me. |
| Structure | Modeling | My parent(s) try to avoid eating junk food or drinking sugary drinks when I am around. |
| Autonomy support | Child involvement | My parent(s) and I decide together how much junk food and sugary drinks I can have. |
| FLASHE, Family Life, Activity, Sun, Health, and Eating. | | |
