# Supplementary material for: Patterns of food parenting practices regarding junk food and sugary drinks among parent-child dyads
Source: Nutr J. 2020 Aug 26;19:91. doi: 10.1186/s12937-020-00610-3 (PMC7448982; doi:10.1186/s12937-020-00610-3)
Supplement: Supplementary file 2 — Additional file 2: Supplementary Table 2. Overall fit statistics for latent class models of parent- and child-reported JS parenting practices. Table contains model fit statistics for the latent class analysis using 1–6 latent classes and based on the 12 parenting practices (6 parent-reported and 6 child-reported) included in the FLASHE surveys. [file 12937_2020_610_MOESM2_ESM.docx]

| **Supplementary Table 2** Overall fit statistics for latent class models of parent- | | | | | | |
| --- | --- | --- | --- | --- | --- | --- |
| and child-reported JS parenting practices | | | | | | |
| **Number of Classes** | **G^2^** | **AIC** | **BIC** | **CAIC** | **aBIC** | **Entropy**^a^ |
| 1 | 6391 | 6415 | 6480 | 6492 | 6441 | 1.00 |
| 2 | 3944 | 3994 | 4130 | 4155 | 4050 | 0.78 |
| 3 | Not well defined^b^ | | | | | |
| 4 | 2923 | 3025 | 3301 | 3352 | 3139 | 0.75 |
| 5 | 2692 | 2820 | 3167 | 3231 | 2964 | 0.76 |
| 6 | Not well defined^b^ | | | | | |
| JS, junk food and sugary drinks; G2, likelihood ratio test; AIC, Akaike Information | | | | | | |
| Criteria; BIC, Bayesian Information Criteria; CAIC, Consistent Akaike Information | | | | | | |
| Criteria; aBIC, adjusted Bayesian Information Criteria. | | | | | | |
| ^a^ Refers to the certainty of model classification; values near 1 indicate high | | | | | | |
| certainty. | | | | | | |
| ^b^ Less than 60% of seeds converged to single solution and/or classes were not | | | | | | |
| clearly distinguished from one another. | | | | | | |
