# Supplementary material for: Patterns of food parenting practices regarding junk food and sugary drinks among parent-child dyads
Source: Nutr J. 2020 Aug 26;19:91. doi: 10.1186/s12937-020-00610-3 (PMC7448982; doi:10.1186/s12937-020-00610-3)
Supplement: Supplementary file 3 — Additional file 3: Supplementary Table 3. Latent class prevalence and item-response probabilities for parent- and child-reported JS parenting practices. Table contains the class prevalence and item-response probabilities for the 5-class solution using latent class analysis on the 12 parenting practices (6 parent-reported and 6 child-reported) included in the FLASHE surveys. [file 12937_2020_610_MOESM3_ESM.docx]

| **Supplementary Table 3** Latent class prevalence and item-response probabilities for parent- | | | | | |
| --- | --- | --- | --- | --- | --- |
| and child-reported JS parenting practices | | | | | |
| **Parenting Practices** | **Complete Influencers** | **Indifferent Influencers** | **Negative Influencers** | **Minimal Influencers** | **Disagreeing Influencers** |
| Class prevalence | 0.28 | 0.21 | 0.20 | 0.18 | 0.13 |
| *Item-response probabilities*^a^ | *Parent-reported* | | | | |
| CC: negative emotions | 0.19 | 0.11 | 0.32 | 0.09 | 0.19 |
| CC: restriction | 0.90 | 0.07 | 0.66 | 0.18 | 0.78 |
| S: monitoring | 0.89 | 0.24 | 0.75 | 0.33 | 0.88 |
| S: availability | 0.86 | 0.26 | 0.25 | 0.82 | 0.67 |
| S: modeling | 0.79 | 0.08 | 0.08 | 0.58 | 0.56 |
| AS: child involvement | 0.75 | 0.05 | 0.41 | 0.27 | 0.65 |
|  | *Child-reported* | | | | |
| CC: negative emotions | 0.22 | 0.13 | 0.37 | 0.11 | 0.11 |
| CC: restriction | 0.92 | 0.01 | 0.70 | 0.27 | 0.09 |
| S: monitoring | 0.88 | 0.05 | 0.77 | 0.34 | 0.15 |
| S: availability | 0.84 | 0.15 | 0.21 | 0.86 | 0.36 |
| S: modeling | 0.80 | 0.04 | 0.16 | 0.67 | 0.17 |
| AS: child involvement | 0.83 | 0.03 | 0.53 | 0.29 | 0.12 |
| JS, junk food and sugary drinks; CC, coercive control; S, structure; AS, autonomy support. | | | | | |
| ^a^ Probabilities reported for agree/strongly agree responses and represent probability of reporting | | | | | |
| agreement with a specific parenting practice given membership in a specific latent class. | | | | | |
